# Pediatric produce prescription initiatives in the U.S.: a scoping review

**DOI:** 10.1038/s41390-023-02920-8

**Published:** 2023-12-04

**Authors:** Hemen Muleta, Laura K. Fischer, Megan Chang, Noah Kim, Cindy W. Leung, Chinwe Obudulu, Kofi Essel

**Affiliations:** 1 General and Community Pediatrics, Children’s National Hospital, DC Washington, USA; 2 Pediatric Hospital Medicine, Children’s Hospital at Montefiore, Bronx, NY USA; 3 Albert Einstein College of Medicine, Bronx, NY USA; 4 The George Washington University School of Medicine and Health Sciences, Washington, DC USA; 5 The George Washington University Milken Institute School of Public Health, Washington, DC USA; 6 Department of Nutrition, Harvard T.H. Chan School of Public Health, Boston, MA USA; 7 Center for Nutrition Policy and Promotion, United States Department of Agriculture, Washington, DC USA; 8 Health Outcomes Organization, Elevance Health, Indianapolis, IN USA

## Abstract

**Background:**

To describe pediatric Produce Prescription (PRx) interventions and their study designs, outcomes, and opportunities for future research.

**Methods:**

A scoping review framework was used to describe PRx interventions published between January 2000 and September 2023. Articles from online databases were uploaded into Covidence. Data on study characteristics, outcomes of interest (health, food insecurity (FI), nutritional and culinary efficacy, and fruit and vegetable (F/V) consumption), and feasibility were extracted. The Mixed Methods Appraisal Tool (MMAT) was used for quality assessment.

**Results:**

19 articles met inclusion criteria. Ten studies were quantitative, five were qualitative, and four used mixed-methods. Interventions included food vouchers (*n* = 14) or food box/pantries (*n* = 5). Four studies allowed food items in addition to F/Vs. Six studies measured changes in FI and five reported a statistically significant decrease. Seven studies measured changes in F/V consumption and five reported a statistically significant increase. One study reported a statistically significant reduction in child BMI *z*-score. Most studies reported high feasibility. Few studies used high-quality methods.

**Conclusions:**

Pediatric PRx interventions show promising potential to reduce FI and improve diet quality and health-related outcomes. Future studies should utilize rigorous study designs and validated assessment tools to understand the impact of pediatric PRx on health.

**Impact:**

- This work offers a summary of programmatic outcomes including retention, redemption, incentives, nutrition education, study design and quality limitations to help inform future work.
- We found positive impacts of pediatric produce prescriptions (PRx) on FI, F/V consumption, and nutritional knowledge and culinary skills.
- More high-quality, rigorous studies are needed to understand the best delivery and design of PRx and their impact on child behavior and health outcomes.
- This work provides support for the need for rigorous studies and the potential for PRx to play a role in multi-pronged strategies that address pediatric FI and diet-related disease.

## Introduction

A high-quality diet rich in fruits and vegetables (F/V) is associated with a decreased risk of chronic disease.^1–4^ However, few children and adults achieve the recommended daily F/V intake.^5,6^ Many factors limit an individual’s F/V intake,^7,8^ including socioeconomic status, food insecurity (FI), and a lack of access to adequate and nutritious food.^9–11^ In 2021, the prevalence of FI in the United States (U.S.) was 10.2%, but higher in households with children (12.5%) and in Black (19.8%) and Hispanic (16.2%) households.^12^ In adults, FI is associated with increased risk of chronic disease such as hypertension, diabetes, and stroke.^13^ While its association with risk of obesity, hypertension, and diabetes has been mixed in children,^13^ FI is associated with higher health care utilization and cost in children and families.^14,15^ It is also hypothesized that childhood FI could contribute to chronic diseases in adulthood, although there is not yet a clear understanding of this relationship. Given the disproportionate burden of FI in children and the rise in chronic conditions, such as diabetes, hypertension, and obesity in children over the past decade,^16,17^ FI and nutrition focused interventions are an important area for research.

Programs addressing FI and nutrition exist in school- and community-based settings.^18–20^ However, as FI and diet-related chronic disease are core medical concerns, interventions integrated within the healthcare setting are critical. According to the National Produce Prescription Coalition (NPPC) Produce Prescriptions (PRx) are “a medical treatment or preventative service for eligible patients due to diet-related health risks or conditions, food insecurity, or other documented challenges in access to nutritious foods, and are referred by a healthcare provider or health insurance plan. These prescriptions are fulfilled through food retailers and enable patients to access healthy produce with no added fats, sugars, or salt, at low or no cost to the patient. When appropriately dosed, PRx interventions are designed to improve healthcare outcomes, optimize medical spending, and increase patient engagement and satisfaction.”^21^ PRx interventions fall under the Food is Medicine framework (or Food as Medicine).^22^ These interventions work within the healthcare systems to offer patients with a diet-related chronic disease risk factor, such as prediabetes or obesity, and who may be at risk or experiencing food insecurity, greater access to produce. Low- or no-cost fresh, frozen, or canned produce and sometimes other food items such as non-perishable healthy staples (i.e. legumes/beans and whole grains) are offered through “incentives” such as redeemable vouchers or directly through provision of food by self-selection or pre-selection via pick-up or delivery. The goal of PRx is to help to prevent, manage, or treat diet-related disease.^21,23,24^ PRx studies have reported positive impacts on food security, health, and food intake among adults,^25–30^ as has been summarized in previously published reviews.^23,31–33^ The potential long-term health benefits of pediatric-focused PRx have not yet been well-studied given the relative novelty of this field.

To our knowledge, there is no published literature that summarizes pediatric-focused healthcare based PRx interventions. Furthermore, the rapid growth of the PRx interventions over the past 2–3 years renders a need for critical review of publications in the field. In particular, evidence for the impact of these initiatives on household FI and family dietary-related behaviors is lacking. This scoping review aims to describe the range of studies, interventions, and outcomes that exist in pediatric PRx interventions in healthcare settings and identify gaps and future directions for the field.

## Methods

A protocol was developed based on the five-stepwise scoping review framework,^34,35^ which included defining the research question, creating search criteria, developing data gathering and analysis procedures, and reporting findings.

Relevant articles were assessed for eligibility using preset inclusion/exclusion criteria. Eligible articles described empirical studies, implementing a PRx in households with children <18 years old (yo); delivered its PRx within the U.S. healthcare system; reported child or adult FI, health, nutritional efficacy, or behavioral outcomes; and were published between January 2000 and September 2023. We also included PRx interventions that were primarily focused on F/V access but offered additional nutritious food items to provide a comprehensive review of interventions targeting F/V intake. Exclusion criteria were as follows: not in English; published before 2000; review articles; conducted outside of the U.S.; exclusively in adults; studies conducted outside of a healthcare system; studies without interventions or interventions that did not include a PRx component; and studies without measured outcomes.

Articles were obtained from online database searches on Pubmed, Scopus, Cumulative Index to Nursing and Allied Health Literature (CINAHL), Cochrane, and Medline. A research librarian assisted in the creation of search terms, which included “food OR produce prescription OR vouchers OR programs OR pharmacy”, “pediatrics OR children OR adolescents”, “health outcomes”, and “food insecurity”. A full list of search terms can be found in Table 1. Articles were uploaded into Covidence (Veritas Health Innovation Ltd, Melbourne, Australia)^36^ for title and abstract screening, full-text review, and final determination of eligibility for data abstraction by two independent reviewers based on the pre-set inclusion/exclusion criteria. Disagreements between screeners were resolved with review by a third screener.

**Table 1:** Search strings used in database searches.

SCOPUS	1) (Food prescription [mesh] OR produce prescription [tiab] OR food voucher [tiab] OR food program* [tiab]) AND (health outcomes* OR food insecurity AND child* OR adolescent*) 2) (“food prescription”), (“food prescription”) AND (child*) 3) (“supplemental nutrition program”) AND (child*)
PUBMED	1) (food prescription program) AND (child OR childhood) AND (obesity) 2) ((food prescription program) AND (nutrition)) AND (adolescent) 3) (food prescription program) AND (adolescent) 4) (supplemental nutrition) AND (adolescent) AND (food prescription) AND (program) 5) (“produce prescription”) AND (childhood) 6) (“fruit and vegetable prescription”) AND (adolescent) 7) (“fruit and vegetable prescription”) AND (children) 8) (“fruit and vegetable prescription”) AND (family) 9) (“produce prescription”) AND (family) 10) (“produce prescription”) AND (obesity) 11) (food) AND (child obesity) AND (prescription) AND (program) 12) (food prescription) AND (child obesity) AND (program)
CINAHL^a^	1) (food prescription) AND (program) AND (childhood or child or children) 2) (food prescription) AND (nutrition) 3) “Food prescription program” AND “children” 4) “Food prescription program” AND “pediatric” 5) “Food prescription program” AND “children” 6) “Food prescription program” AND “fruits and vegetables” AND “children” 7) “Food prescription program” AND “voucher” AND “children” 8) “Food prescription program” AND “voucher”
COCHRANE	1) (food prescription program) AND (child OR childhood) AND (obesity) 2) ((food prescription program) AND (nutrition)) AND (adolescent) 3) (food prescription program) AND (adolescent) 4) (supplemental nutrition) AND (adolescent) AND (food prescription) AND (program)
MEDLINE	1) “Fruits/ and Vegetables/” AND “Child/” AND “food prescription or Food/”

^a^*CINAHL* Cumulative Index to Nursing and Allied Health Literature.

A data abstraction form and data reporting were guided by the Preferred Reporting Items for Systematic reviews and Meta-Analyses extension for Scoping Reviews (PRISMA-ScR) guidelines.^37^ Data were abstracted to describe the study characteristics (study and intervention design, setting, inclusion/exclusion criteria. When reported by articles, the maximum incentive amounts offered per intervention were calculated using the reported amount, frequency, and duration of incentive offered), feasibility, and changes in health, FI status/severity, nutritional efficacy, and behaviors. Two independent reviewers extracted data for each article and an independent third reviewer reconciled discrepancies. A quality assessment of included studies was conducted using the Mixed Method Appraisal Tool (MMAT),^38^ which uses five criteria related to the appropriateness of the study design, representativeness of the population, adherence to the stated intervention, completeness of outcome data, and interpretation of the results to assess the methodological soundness of studies. Thus, each study received a rating on a scale of 0 to 5 corresponding to the quality of the study with respect to the stated primary outcome. Two independent reviewers evaluated each article and met regularly to come to a consensus on the quality rating for each study.

## Results

### Article screening

**Fig. 1:**
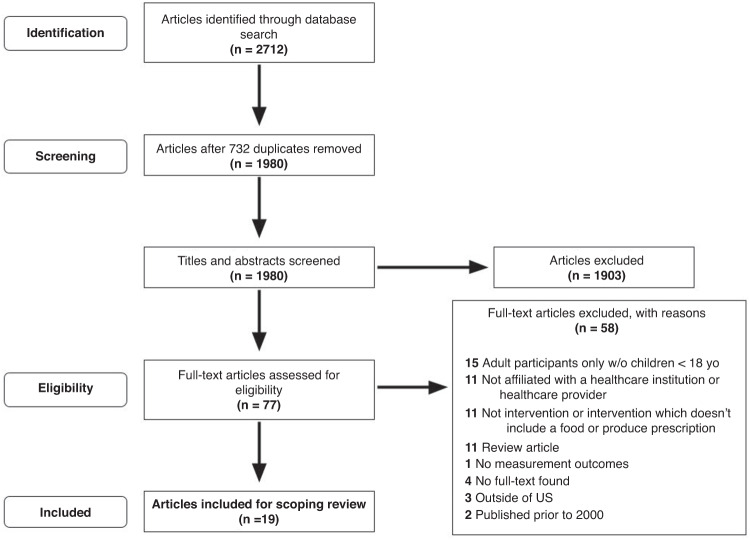
PRISMA flow diagram of articles in the scoping review. Figure 1 shows the flow from article identification to selection. The original database search yielded 2711 records. After duplicates were removed, there were 1980 unique citations. After screening by title and abstract, there were 77 eligible, full-text articles. Upon assessing eligibility, it was found that 59 full-text articles were not eligible: 15 did not include children, 11 had no affiliation with a healthcare institution or provider, 11 did not include a food or produce prescription, 11 were review articles, 4 did not have a full-text available, 3 were published outside the U.S., 2 were published prior to 2000, and 1 did not have measurement outcomes. A total of 19 studies were included for scoping review.

1980 articles underwent title and abstract screening of which 77 underwent full-text screening and 19 articles met eligibility criteria (Fig. 1), Of these 19 articles, four described results from one intervention.^39–42^ All 19 studies were published after 2014; the majority (*n* = 11) were published between 2020–2023.^39,40,43–51^

### Study design, setting, and populations

**Fig. 2:**
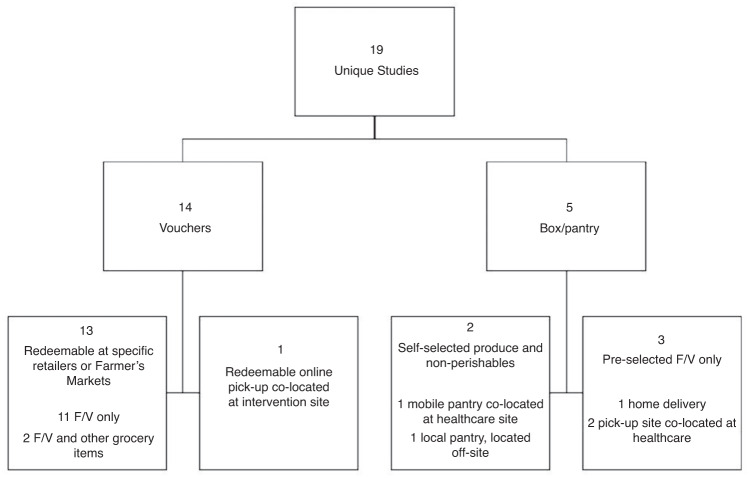
Description of produce prescription intervention types. There were a total of 19 interventions evaluated, intervention models fell into two categories: voucher programs (*n* = 14) or box/pantry programs (*n* = 5). Thirteen voucher programs utilized farmers’ markets (FM) or retailers and one used an online produce market. Two voucher programs allowed redemption for other foods in addition to produce. Among box programs, two allowed self-selected produce and non-perishable items and three programs offered pre-selected F/Vs only. One provided delivery services and the rest were pick-up.

See a summary of intervention delivery types in Fig. 2 and results in Table 2. Ten studies were quantitative,^39–41,45,46,48,49,51–53^ five were qualitative,^42,44,50,54,55^ and four used mixed methods.^43,47,56,57^ Of the 14 quantitative or mixed methods studies, 13 were longitudinal and one was cross-sectional.^40^ There were no were randomized control trials (RCTs), two studies utilized control groups to compare outcomes,^40,52^ and the remainder were pre-post comparisons. One study reported the aggregated outcomes from data collected across 9 program sites, but only three sites enrolled children, so we will only report on the pediatric programs and outcomes reported in this multi-site study.^51^ Fourteen took place in primary care settings, two in a school-based health system,^43,54^ two in subspecialty clinics,^50,55^ and one did not specify.^51^ In all but two cases,^50,54^ patients and families were directly referred to the program by a healthcare provider. In most cases this was a primary care provider (Pediatrician, Nurse Practitioner) and in some cases it was ancillary clinic staff or allied health professionals (Community Health Worker or Dietitian/Nutritionist). Eleven studies were conducted within urban settings,^39–42,46–48,50,52,54,56^ three within rural settings,^43–45^ and five did not specify.^49,51,53,55,57^ The number of participants ranged from four^55^ to 1,817^51^ with a geometric mean of 79. Twelve studies had health risk factors, FI status/severity, or income related inclusion criteria.^43–49,51,53,55–57^ Seven did not have health- or income-specific inclusion criteria.^39–42,50,52,54^ All studies included both adult and child participants. Children’s age ranges were <1 yo (*n* = 1),^52^ <6 yo (*n* = 3),^45,47,48^ 2–18 yo (*n* = 7),^39,41,44,46,51,53,57^ 0–15 yo (*n* = 1),^54^ and any age (0–18 yo) (*n* = 7).^40,42,43,49,50,55,56^

**Table 2:** Characteristics of Produce Prescription (PRx) articles included in scoping review.

First Author Year	Setting State Healthcare setting Urban/rural	Primary aim	Participants Child age Inclusion criteria Sample size	Intervention Incentive amount and type Incentive frequency and duration Nutrition education offered Retention and Redemption	Study Design and Assessment Tools Study type Data collection methods and Assessment tools	Reported Outcomes	MMAT^u^
Abel 2022^46^	NY PCC^a^ in AC^b^ Urban	Examine the relationship between PRx^f^ redemption and 1) FI^g^ 2) sociodemographics 3) nutrition-related health measures	>7 years old Any nutrition concerns *n* = 242	$10–20 F/V vouchers to FM Once over 5 months NNE^k^ Retention: NA^l^ Redemption: 49.3%	Quantitative Retrospective cross-sectional survey and med chart review	Program was feasible and reached its target audience. Those with FI and elevated Hemoglobin A1c were more likely to redeem prescriptions.	3
Aiyer 2019^56^	TX PCC, FQHC^c^ Urban	Examine the feasibility, perceptions, and impact of a collaborative food prescription program in an area with a high rate of FI.	All ages Household FI *n* = 242	30-pound bag of F/V, and 4 “healthy” non-perishable items Twice monthly for 6 months Nutrition education booklets Retention: 17% (42/242) Redemption: 35–39%	Mixed-method, cohort Pre/post surveys, including validated screener HVS^p^ and qualitative interviews	Program was acceptable and feasible among participants and providers. Participants reported positive changes in food shopping behavior, decrease in FI status**^**, and increase in nutritional knowledge and attitudes towards healthy foods.	2
Brown 2022^50^	PA Subspecialty clinic in AC Urban	Examine the PRx intervention’s ability to reach families with FI without eligibility criteria, and caregiver experiences and preferences for programming	All ages No health/income inclusion criteria by design *n* = 31	“Box” of F/V Weekly for 3 months Recipe card and information via text messages Retention: NA Redemption: 33% (average redemption of 4 out of 12 weekly offerings)	Qualitative, Key informant interviews and baseline demographics	Program was acceptable to caregivers due to efficiency and ease, quality of F/V, and interactions with program staff. Participants reported improved attitudes towards produce and confidence in buying produce, increased exposure, interest, and acceptance of F/V	5
Burrington 2020^43^	NY SBHC^d^ Rural	To promote lifestyle dietary changes via a combined PRx and cooking and nutrition education classes	All ages BMI > 85% and income *n* = 39	$15–25 F/V vouchers to online market Weekly for 5 months EFNEP^m^ and cooking classes Retention: 80% Redemption: 94%	Mixed-method, cohort Pre/post surveys without specifying validated tools, and qualitative interviews	Authors reported increased consumption of F/V by parents and children, increased family time preparing home cooked meals, confidence in cooking skills and use of F/V in meals. Authors also reported decreased FI.	2
Esquivel 2020^44^	HI PCC, FQHC Rural	Examine feasibility of a community based pediatric PRx	2–17 years old Growth concerns (BMI < 5% or >85%) *n* = 125	$24 F/V vouchers to FM Monthly for 3 months NNE Retention: 27% Redemption: 63% partially redeemed, 17% fully redeemed	Qualitative Semi-structured phone interviews	Program was feasible and acceptable. Pediatrician involvement helped build trust and encouraged participation in the program. Participants reported positive impacts on diet and activity, access to F/V, and interest in FM.	2
Fischer 2022^47^	DC PCC in AC Urban	Examine feasibility and explore the impact of PRx on FI and F/V^h^ intake and to guide the design of future iterations of the program.	0–5 years old FI and diet-related chronic disease risk *n* = 25	8-pound boxes of F/V Delivered twice monthly for 12 months Written & video recipes, cooking classes Retention: 60% Redemption: 75%	Mixed-method, cohort Pre/post surveys using validated FFQ^q^, USDA 6-item FI, and Cooking Matters surveys; and semi-structured qualitative interviews	Program was feasible and acceptable. Participants reported increase in daily fruit intake in children^* and vegetable intake in children**^**, increase in daily F/V intake of adults**^**, and child and adult experimentation with new foods. Decreases in FI severity**^** Participants learned food preparation skills, and children were more engaged in cooking/preparing F/V.	4
Forbes 2019^57^	PA PCC in AC, hospital NS^e^	Examine how participation in a more comprehensive PRx might change behaviors and perceptions about healthy eating.	>5 years old Risk of chronic illness or metabolic disease, and food access concerns *n* = 10	$40 F/V vouchers to FM Four sessions, over 6 weeks Nutrition teaching sessions and coaching by medical students based on USDA^n^ MyPlate Retention: 90% Redemption: NR^o^	Mixed-method, cohort Pre/post surveys using validated BRFSS^r^, IPAQ^s^, and CookWell surveys; and qualitative semi-structured interviews by phone	Increased consumption of F/V, decrease in nutritionally poor foods, increase in physical activity. Increased confidence in cooking.	3
George 2016^55^	PA Weight management clinic in AC NS	Examine feasibility, strengths, and limitations of a PRx.	All ages Income and BMI *n* = 4	$50 F/V vouchers to FM Four sessions over 2 months Nutrition coaching by medical student based on “healthSLAM” Retention: 50% attended all sessions Redemption: NA	Qualitative Focus group interviews	Program was feasible, with positive responses from caregivers, medical providers, and vendors. Increased involvement of children in meal preparation; children were more likely to eat produce they chose from the market or picked from the garden. The program increased access to F/V.	4
Hager 2023^51^ Only reporting results from pediatric sites	CA, TX, FL, NY, ID	To test the hypothesis that PRxs improve participant FV intake, food insecurity, and health outcomes (self-reported health status, BMI^i^, BMI z-scores).	2–18 years old BMI and FI, or community eligibility *n* = 1817	$60 to $300 (median= $112 (IQR, $85–$133) vouchers to retailers for F/V Monthly for 6–9 months In-person or online nutrition education classes Retention:NR Redemption: 77.1%	Quantitative, cohort Pre-post self-report surveys: validated dietary questionnaires, health status Medical chart review: weight, height	F&V intake increased in children^^*^ Increase in self-reported health status in children^^*^ Non-significant decrease in Child BMI z-score^^^	4
Jones 2020^45^	AZ, NM, UT PCC, hospital Rural	Examine the impact of a PRx on changes in health behavior, BMI and FI of participating children.	≤ 6 years old FI risk *n* = 212	$1/day/person up to $5/day vouchers to various retailers (FMs, stores, & trading posts that met criteria) for F/V and culturally-relevant foods Monthly for 5–6 months Individual or group sessions w/ trained staff based on “Healthy Habits, Healthy Homes” Retention: 57% Redemption: NR	Quantitative, cohort Pre/post survey, using validated BRFSS and USDA 6-item FI	Decrease in BMI in children who had overweight/obesity^* Child F/V consumption increased^*, proportion of children meeting AAP^t^ recommendations for F/V increased^*, improvement in physical activity, sleep, and screen time**^** Decrease in FI status*	4
Orsega-Smith 2020^49^	DE Private PCC NS	Examine a PRx program, describe outcomes after 1 year.	All ages FI, income, and BMI *n* = 41	15–25 pounds box of F/V from a mobile pantry at clinic Monthly for 12 months Cooking demonstrations Retention: NR Redemption: NR	Quantitative, cohort Pre/post surveys without specifying validated tools	Increase in daily servings of F/V in adults^*, increase in daily servings of fruit in children^*, increase in daily servings of vegetables in children**^**. Increased access to produce.	2
Ridberg 2019^53^	DC, ME, MA, NM, RI, NY PCC, FQHC NS	Examine changes in household FI associated with participation in a PRx.	2–18 years old BMI *n* = 578	$0.50 to $1.00/person/day F/V vouchers to FM Per monthly clinic visit for four to six months Nutrition education classes by Wholesome Wave Retention: NR Redemption: 54%	Quantitative, cohort Pre/post survey, using 5-item scale developed from USDA 18-item FI survey	Decrease in FI severity^*, those with highest clinic visits had greatest reduction in FI score^*	4
Saxe-Custack 2018^42^	MI PCC in AC Urban	Examine caregiver perceptions of an urban pediatric clinic co-locating with a FM^j^, experiences with a PRx and perceived impact on child consumption of F/V.	No age specified No health/income inclusion criteria *n* = 32	$10 F/V vouchers to FM or vendor-prepared bag of F/V Per clinic visit, duration unknown NNE Retention: NR Redemption: NR	Qualitative Semi-structured in-person or phone interviews	Perceived increases in child consumption of produce due to participation in the program. Perceived increases in access to produce due to participation in the program.	5
Saxe-Custack 2019a^41^	MI PCC Urban	Examine changes in the consumption of whole fruit following six months of exposure to the PRx.	7–18 years old No health/income inclusion criteria *n* = 114	$15 F/V vouchers to FM or a mobile market Per clinic visit, frequency and duration not specified NNE Retention: 90–99% Redemption: NR	Quantitative, cohort Pre/post survey, using validated FFQ	Child increase in daily serving of whole fruits^*, increase in consumption of vegetables**^**, and decrease in fruit juice consumption**^**.	4
Saxe-Custack 2019b^54^	MI SBHC Urban	Examine caregiver perceptions of the PRx.	0–15 years old No health/income inclusion criteria *n* = 37	Six individual $5 vouchers to FM redeemable for various food items Given once at end of school year NNE Retention: NR Redemption: 53%	Qualitative Semi-structured phone interviews	Increased access to produce and child participation in food selection.	5
Saxe-Custack 2020^40^	MI PCC in AC Urban	Examine the association between participation in a PRx and FM shopping.	All ages No health/income inclusion criteria *n* = 157	$10 F/V vouchers to FM Per clinic visit, frequency and duration not specified NNE Retention: NR Redemption: NR	Quantitative, cross-sectional Self-report surveys	Participants were more likely to visit farmers’ markets than non-participants^*	4
Saxe-Custack 2021^39^	MI Private PCC Urban	Examine preliminary effectiveness of a PRx.	6–18 years old No health/income inclusion criteria *n* = 244	$15 F/V vouchers to FM or local mobile market At every clinic visit for one year NNE Retention: NR Redemption: NR	Quantitative, cohort Pre/post survey, using validated tools USDA 6-item FI, USDA Child Food Security Survey Module, and FFQ	Child increase in daily intake of vegetables, whole grains, fiber, and dairy^*, increase in consumption of fruits**^**, increase in all children having at least one cup of F/V daily compared to baseline^* Decrease in household and child FI severity^*	4
Watt 2015^52^	TX Public PCC Urban	Examine process and outcome metrics of a primary care-based PRx targeting low-income Hispanic women.	Perinatal period up to 6 months old No health/income inclusion criteria *n* = 61 [intervention *n* = 32, control *n* = 29]	F/V vouchers to a FM, amount not specified Frequency of distribution not reported, over 24 weeks Nutrition and cooking classes based on Healthy Active Living for Families program Retention: 50% and 74% Redemption: 16.6%	Quantitative, cohort with a non-intervention control group Pre/post survey, using validated tools FFQ, Developmental Questionnaire, ENRICHD Social Support Instrument, Cohen Perceived Stress Scale, Patient Health Questionnaire	Increase in consumption of F/V^* and other healthy food^*, and reduction in chips/crackers and fat^* in the intervention. Increase in physical activity^* in the intervention group. Reduction in depression screening scores in intervention^*. No significant difference in baby’s weight or maternal weight**^**, weight/length percentile**^**, maternal blood pressure**^**, or breastfeeding rate**^**.	5
Woo Baidal 2022^48^	NY PCC in AC Urban	Examine reach, feasibility, and retention of a PRx and explore characteristics correlated with retention and attrition.	<6 years old Household FI *n* = 50	Box of pantry items (F/V and non-perishables) equivalent to 12 meals/ family Twice monthly for six months Cooking demonstrations from nutritionist Retention: 68% Redemption: NR	Quantitative, cohort Pre/post self-report surveys	Program reached target population	4

^a^*PCC*, Primary Care Clinic;

^**b**^*AC*, Academic Center;

^c^*FQHC*, Federally Qualified Health Centers;

^d^*SBHC*, School Based Health Centers;

^e^*NS*, Not Specified;

^f^*PRx*, Produce Prescription Program;

^g^*FI*, Food Insecurity;

^h^*F/V*, Fruits and Vegetables;

^i^*BMI*, Body Mass-Index;

^j^*FM*, Farmers’ Market;

^k^*NNE*, No Nutrition Education;

^l^*NA*, not applicable;

^m^*EFNEP*, Expanded Food and Nutrition Education Program;

^n^*USDA*, U.S. Department of Agriculture*;*

^o^*NR*, Not reported*;*

^p^*HVS*, Hunger Vital Sign;

^q^*FFQ*, Food Frequency Questionnaire;

^r^*BRFSS*, Behavioral Risk Factor Surveillance System;

^s^*IPAQ*, International Physical Activity Questionnaire;

^t^*AAP*, American Academy of Pediatrics;

^u^*MMAT*; Mixed Method Appraisal Tool.

**^**statistical analysis performed; *p-value < 0.05 (statistically significant result).

### Incentive models

The intervention incentive models fell into two categories: voucher programs (*n* = 14),^39–46,51–55,57^ or box/pantry programs (*n* = 5).^47–50,56^ Thirteen voucher programs utilized farmers’ markets (FM) or retailers while one used an online produce market.^43^ All voucher programs allowed redemption for produce, though two programs allowed redemption for other food sold at FM locations, like meats, cheeses, baked goods,^54^ or culturally significant foods like blue cornmeal or dried steam corn.^45^ Among box programs, two allowed self-selected produce and non-perishable items^48,56^ and three programs offered pre-selected F/Vs only.^47,49,50^ One provided delivery services^47^ and the rest were pick-up only.

### Distribution of incentives

There was wide variability in the amount, duration, and frequency of incentive distributions. Among voucher programs, the lowest distribution amount was a one-time voucher of $10 or $20.^46^ The highest incentive amount across all voucher interventions was $300 per month for 6–9 months, which would equal a theoretical maximum value of $2,700.^51^ The remaining voucher interventions were calculated to have a range of maximum incentives from a one-time distribution of $30^54^ to $900 over 6 months^43,45^ per household. Four interventions varied the voucher amount by family size^43,45,51,53^ and one varied voucher amount by FI status.^46^ Among box programs, three studies^47,49,56^ reported pounds of produce offered, which ranged from 8 to 30 pounds per distribution. One study described a “box” of produce per distribution^50^ while another study indicated the amount as “12 meals per household” per distribution.^48^ The range of calculated maximum amounts of food offered by box programs was 192 pounds over 12 months^47^ to 360 pounds over 6 months^56^ per household. One box program varied the food amount by family size.^48^ Among all studies, distribution frequencies occurred once during the intervention (*n* = 2),^46,54^ weekly (*n* = 2),^43,50^ twice monthly (*n* = 3),^47,52,56^ monthly (*n* = 5),^44,45,49,51,53^ and at each clinic visit (*n* = 4).^39–42^ Duration of programs ranged from 1.5 months^57^ to one year,^39,47,49^ with an average duration of six months.

### Education

Twelve articles described a nutritional education component in their intervention.^43,45,47–53,55–57^ These included cooking classes,^43,47–49,52^ nutrition classes,^45,51,53^ booklets,^56^ individual coaching,^55,57^ videos,^47^ and written recipes.^47,50^ Four studies reported on frequency and/or duration of education, which corresponded to 24 h total,^47^ 16 h,^52^ monthly sessions,^53^ and four total sessions.^55^ Incentive redemption was explicitly tied to education session attendance in two studies,^45,48^ otherwise, education was not mandatory for incentive redemption. Healthcare clinic staff or providers (Clinician, Nutritionist or Health Educator) were involved in delivering nutrition education in four^47,48,52,53^ out of the 12 programs that offered nutrition education.

### Behavioral outcomes

Fifteen studies reported on food consumption patterns, food purchasing and cooking habits, and physical activity.^39–45,47,49–52,55–57^ Twelve studies reported F/V intake with qualitative (*n* = 3) or quantitative (*n* = 9) tools. Among the quantitative assessments of F/V intake, two studies^43,49^ used non-validated tools, and seven^39,41,45,47,51,52,57^ used validated instruments. Statistical testing was conducted in five studies.^39,41,45,47,51^ All five showed significant increases in child F/V intake, with increases in fruit intake of 30% from 0.6 to 0.8 servings per day (PD)^41^ and 43% (from 0.8 to 1.3 cups PD),^47^ a 33% increase in vegetable intake (from 0.7 to 0.9 servings PD),^39^ and a 31% and a 7.5% increase in total F/V intake (from 5.2 to 6.8 cups PD^45^ and from 3.47 to 3.73 cups PD,^51^ respectively). Two studies statistically tested and reported the change in adult caretaker F/V intake. One found a significant increase in adult vegetable intake (from 2.22 to 2.44 servings PD) and fruit intake (from 2.05 to 2.46 servings PD),^49^ while the other found a non-significant increase in adult F/V intake.^47^ One study reported families were more likely to shop at an FM in the month following the intervention compared to non-participants.^40^ Four studies reported changes in physical activity outcomes,^44,45,52,57^ in one study the increase was statistically significant.^52^

### Food insecurity outcomes

Eleven studies^39,42–45,47,49,53–56^ evaluated changes in FI or food access; six used non-validated or qualitative assessment methods^42–44,49,54,55^ and five used validated FI instruments.^39,45,47,53,56^ Of these five, four^39,45,47,53^ measured FI severity using variations of the USDA Household Food Security Survey^58^ while the fifth^56^ used the Hunger Vital Sign (HVS) screener.^59^ Overall, these studies reported increased access to food, including F/V, and improved FI status of households. Among the five that utilized validated tools and conducted statistical testing, four reported significant improvements – two in FI status^45,56^ and two in FI severity.^39,53^ There were reported reductions in household FI status by 94% (100% at baseline to 5.9% at 6 months)^56^ and 17% (82% at baseline to 65% at 6 months)^45^ and reductions in FI severity by 55% (1.96 at baseline to 0.87 at 12 months)^39^ and 12.5% (from 0.72 at baseline to 0.81 at 6 months, this tool scored food security, so an increase is a reduction in FI severity).^53^ One study reported a “dose”-response effect on FI severity, indicating those with higher intervention participation (5–6 visits out of 6) had a greater reduction in FI severity than those who only attended 1–2 visits out of 6 (effect size β = 0.07).^53^ One reported non-significant improvements in FI severity.^47^ Only one study measured longitudinal change in FI as reported by the child and found a statistically significant reduction in their modified FI score from 1.88 at baseline to 1.04 at 12-months.^39^ Hager et al. reported household FI but it was not possible to disaggregate FI data in households with children from all households.^51^

### Nutritional & culinary efficacy outcomes

Nutritional and culinary outcomes, including confidence, skills, knowledge, and attitudes towards the preparation, storage, consumption, and purchasing of foods, were reported by six studies.^43,44,47,50,56,57^ Three studies utilized qualitative measures^43,44,50^ and three utilized mixed measures,^47,56,57^ with two using validated questionnaires.^47,57^ There were no statistical analyses of nutrition and culinary outcomes, though all studies reported improved cooking skills and increased confidence in cooking, utilizing produce, and following recipes.

### Health outcomes

Only three studies measured biometrics and health outcomes such as weight, body mass index (BMI),^45,51,52^ and health status^51^ in children. One study showed a statistically significant decrease in BMI z-score (95.6 to 73.1) in children who were classified as having overweight or obesity at baseline.^45^ The other studies found no significant effect of the intervention on child weight.^51,52^ One study measured change in health status on a five-point scale (poor, fair, good, very good, excellent), and found a statistically significant likelihood of improving one level from baseline after program participation (for example going from poor to fair, or fair to good).^51^ One study measured change in depression screening scores in adult mothers and found a reduction in scores in women in the intervention arm and no significant change in scores in women in the control arm.^52^

### Feasibility

Feasibility was measured primarily by enrollment of target population, perceived positive impact, and satisfaction in interventions both by adult participants and medical providers. All seven studies which looked at feasibility reported that their interventions were feasible and acceptable within the intended population.^44,46–48,50,55,56^

### Retention and redemption

Retention rates ranged from 27%^44^ to 4.5%.^41^ Redemption ranged from below 20%^52,56^ to 80% or greater.^43,55^ Retention and redemption reporting methods were not standard across studies and some studies did not explicitly report on retention or redemption rates of their participants.

### Summary of qualitative

Qualitative responses revealed decreased financial hardship and increased access to healthy affordable food.^42,44,47,50,54,56^ Perceived improvements in F/V consumption, number of home cooked meals, and culinary skills and shopping habits were common.^42–44,47,50,54,55^ In addition, household-level attitude changes towards healthy eating, increased family time, and increased involvement of children in the cooking process were reported.^43,44,47,50,54^ Qualitative statements also captured site-specific barriers to participation, concerns over sustainability of perceived impacts after the program ends,^57^ and the importance of healthcare-based delivery of programs as a motivation for participation.^44^ Studies which evaluated feasibility through stakeholders including families, food vendors, and program assistants showed favorable responses towards their programs.^55–57^

### Quality appraisal and limitations

The majority of studies were feasibility or pilot studies with small sample sizes, which limits their generalizability and ability to assess the impact of an intervention. MMAT results, as presented in Table 2, reflect acceptable quality with regard to feasibility outcomes. However, with respect to outcomes related to FI and F/V intake, there was a lack of high-quality methods, including not using validated assessment tools, having incomplete data, not controlling for confounders in the design and analysis, and lacking or inappropriate statistical methods.

#### Lessons Learned

We have highlighted some considerations for implementation of pediatric PRx interventions in Table 3.

**Table 3:** Considerations for Implementation of a Pediatric Produce Prescription (PRx) Intervention.

Incentive Design	Referral Stream	Engagement	Evaluation and Dissemination
Ease of redemption: Attempt to limit constraints of redemption or fulfillment around transportation and convenience “Dose” of incentive: Consider the appropriate size and duration of incentive Consider varying size of incentive to size of family and current inflation impacting food prices Consider length of intervention, balancing family needs and program goals Provision of incentive and education: Partnerships with community organizations may facilitate distribution, sustainability, and education but may require training, healthcare certifications, and supervision	Streamline referrals: Referral through a clinical healthcare provider (*MD, NP, PA, RDs,* etc.) is generally feasible with training and proper support Involve additional clinic staff (*social work, community health workers, front desk, admissions* etc.) in the referral process for better alignment and to distribute responsibilities amongst healthcare team Trust is a factor: Build off pre established trust for clinicians to improve family’s willingness to participate and engage in a PRx	Deliver an enjoyable, respectful, and meaningful program: Prioritize convenience of redemption and educational programming Consider culturally tailored programming and racial/ethnic congruence in marketing to respect target population norms and beliefs Provide an educational component that includes the whole family unit Consider experiential learning opportunities (i.e. teaching kitchens, store/market tours) to support longitudinal learning amongst participants Consider providing ancillary tools to facilitate use of novel foods (*cooking ingredients, equipment,* etc.) Consider legitimate family limitations (*bandwidth, literacy, competing priorities,* etc.) Provide participants with consistent, timely, friendly communication with program staff	Choose appropriate outcome measures: Match outcome metrics to target population (e.g. *measure clinical outcomes when enrolling pediatric patients who present with diet-related disease risk at baseline*) Conduct rigorous assessments: Use validated pediatric assessment tools to measure food insecurity and fruit and vegetable intake Engage a multidisciplinary team to undertake program evaluation and research Request funding for evaluation and dissemination support when creating program budgets Include qualitative methods: Explore the lived experience of participants through qualitative interviews and analysis. Use findings to improve interventions Report program outcomes: Clearly describe programmatic outcome metrics (*rate of redemption, rate of retention, rate of participation,* etc.) using methods described by others Make findings publicly-available: Present outcomes via publication in open-source peer-reviewed journals and white/grey papers, and via conference abstracts, workshops, community meetings

## Discussion

To our knowledge, this is the first scoping review to describe pediatric PRx interventions. There is a rising interest in pediatric PRx, which coincides with the recent national traction that FI, food inequity, and nutrition insecurity have received as major public health concerns and policy priorities in the U.S.^60^ Our work adds to previous reviews of PRx interventions by incorporating recently published studies and focusing on pediatric and caretaker outcomes related to FI, food intake, nutrition and culinary efficacy, and the family perspective. Additionally, it offers a summary of programmatic outcomes of retention, redemption, incentive amounts, nutrition education, and study design and quality limitations to help inform future work. Overall, the studies reviewed reported positive impacts of PRx towards FI, consumption of F/V, nutritional knowledge and culinary skill in households with children. However, more high-quality, rigorous observational cohort and RCT studies need to be undertaken to understand the best delivery and design of PRx interventions and their impact on behavior and health outcomes. This scoping review provides support for the need for further rigorous study and the potential for PRx to play a role in multi-pronged strategies that address FI and diet-related disease in children.

The emergent nature of pediatric PRx is reflected in the prevalence of pilot feasibility and acceptability studies utilizing non-randomized experimental designs. These studies provide a foundational understanding of the various design and implementation strategies of PRx interventions and explore their potential impact on FI and nutrition-related behavior and knowledge. They also provide a rich qualitative perspective of PRx participant experiences, which can help to define the priorities, needs, and assets of the individuals and communities for whom such interventions are being implemented. There was a wide diversity of study settings, a small number of studies, and lack of rigorous methods, limiting the ability to make generalizations about optimal PRx delivery models from this data. The review explored the relationship between PRx intervention characteristics (incentive amount, type, duration; retention and redemption; offering nutrition education, and assessment methods) and FI and produce intake outcomes but found no obvious patterns. As such, further studies are needed to determine the most efficacious intervention design. The optimal delivery and design of PRx interventions are also likely to differ based on the clinical context and the needs of the target population. Future PRx interventions should determine feasibility and efficacy within specific populations, focusing on strengths, challenges and cultural norms that impact participation and outcomes. The research highlighted herein suggests that increasing accessibility to healthy food by overcoming barriers related to transportation, cost, knowledge, and skills is an important part of interventions that positively impact healthy eating and purchasing patterns. Other research suggests prioritizing cultural considerations in food provision and educational programming is also important.^61^

An important next step in the research continuum for pediatric PRx is utilizing more rigorous study designs such as RCTs that include robust program evaluation methods, validated measurable outcomes, and sufficient sample sizes to detect statistically and clinically significant behavioral and health changes. While there are important ethical considerations to randomizing families with children for critically needed nutritional resources, RCTs can be conducted equitably with thoughtful methodology. For example, RCTs could explore PRx interventions with different frequencies, durations, delivery methods, produce quantities, participant choice, and various nutrition education components to better understand the efficacy of various PRx modalities and moderating factors. Programmatic outcomes such as retention and redemption rates should be reported to better understand the programmatic success and “dose” of produce prescription being provided.

While food as medicine approaches such as PRx are often used to treat disease in adult populations, a sole focus on treatment of disease may limit the potential impact of these interventions in children. The studies reviewed support the use of pediatric PRx as primarily preventive of adverse long-term health outcomes in children who are at-risk of diet related disease, while also addressing acute nutritional needs. PRx interventions offer an opportunity to impact the developmental trajectory of children by exposing them to a wide variety of tastes and flavors early in life, which promotes healthy eating behaviors later in life.^62^ Children typically have a slower onset of observable clinical abnormalities and visible signs of disease, however preventing diet-related, chronic disease through PRx may provide cost savings within the healthcare system in these future adults.^63,64^ Pediatric population health has the potential for impacts on a national level, as these current students will soon become workforce adults and eventually become utilizers of Medicare, so behavior changes now are perhaps incredibly powerful for long-term healthcare implications. As such, prevention-related markers, such as micro-environmental, self-efficacy, and behavioral changes, including increased F/V intake, nutritional knowledge gained, and improved food security, which are predictive of long-term health outcomes, may be highly appropriate outcomes to assess the impact of pediatric-focused PRx within a relatively healthy, resilient, and young population and long-term, cohort follow-ups in increments of 5 years would be beneficial. However, the choice of measured outcomes to assess impact, whether clinical metrics or behavioral or environmental proxies of health, will depend on the target population, program goals, duration of the study, and capacity of the research team.

Although some of the articles reviewed measured these proxy outcomes, it is difficult to compare across studies due to the heterogeneity of study designs and assessment tools used. The articles examined utilized three different methods to assess household FI: qualitative interviews, HVS screener, or variations of the USDA food security screener. Studies that utilize the HVS or qualitative tools are not able to determine severity of household FI, making cross-study comparison of the impact of PRx on FI depth difficult. Additionally, PRx interventions may reduce the severity of household and child-level FI rather than eliminate FI, making longitudinal assessments of household and child-level FI severity an important consideration of their evaluation. Standardized measurements are also needed for diet-related outcomes. The studies in this review that reported diet-related outcomes utilized a mix of qualitative, non-standardized single items, and standardized validated questionnaires. Previous adult PRx systematic and scoping reviews have also described difficulty encountering heterogeneous outcome measurements.^32,65^ A major step toward rigorously evaluating PRx interventions came with the 2018 Farm Bill, which funds the Gus Schumacher Nutrition Incentive Program (GusNIP). GusNIP attempts to standardize evaluation outcomes with shared metrics across studies.^66^ Forthcoming data will help identify optimal evaluation strategies that balance participant burden with the need for high-quality data and provide valuable insight into the impact of these programs. Future studies should consider using the free, validated, standardized tools identified by GusNIP to assess severity of FI and changes in F/V intake, in addition to any other stated primary outcomes, so comparison across studies can be done. Many PRx interventions are funded by philanthropic organizations in community health settings and may have little or no in-house technical support for evaluation. However, it is critical for PRx interventions to conduct program evaluation and research using standardized assessment methods and to disseminate results, which may require a multidisciplinary team of community, academic, and clinical collaborators and study budgets should reflect adequate funding for these efforts.

While interventions specifically addressing populations participating in FNPs were not included in this review, there is well-established evidence that these programs can improve FI, decrease poverty, and improve health outcomes.^67^ Although supplemental, these programs often comprise a major portion of family food budgets and remain inadequate for addressing the needs of families struggling with FI,^68,69^ leading to early benefit exhaustion associated with reduced perceived healthfulness of diet^67,70^ and maladaptive eating patterns.^71^ Furthermore, populations that face FI and diet-related chronic illnesses are often more likely to experience additional social risk factors, so effective strategies must continue to advocate for policies that comprehensively address social inequities related to the income and wealth gap that can co-occur in families with FI.^72^ PRx interventions can be one part of a multi-pronged approach, in addition to other policies aimed at enhancing the purchasing power of families with limited resources, including assistance for better income, housing, and employment opportunities, to buffer complex and multifactorial social determinants of health.^73^

Future studies should focus on exploring the impact and sustainability of PRx interventions within healthcare systems. Some barriers to the sustainability of PRx interventions include the cost, complexity, and capacity of integrating PRx within existing clinical settings.^74^ Although these interventions may require a large initial investment, they may provide long term healthcare cost savings. Additionally, the clinician-patient relationship may be enhanced by clinicians offering PRx as a resource for families with FI. Offering resources in response to positive clinical screening may serve as a “trust catalyst” when engaging with patients about social determinants or sensitive diet-related chronic disease topics.^75^ The success of integrating medically tailored meals (MTMs) within major state and private insurance systems serves as an example for the potential future of PRx at-large.^76^ To demonstrate impact and sustainability of pediatric PRx interventions, the field must undertake hypothesis-driven studies, powered to measure clinically significant changes in FI, and health-related behaviors and health outcomes. Additionally, future studies should conduct cost-effectiveness analysis with respect to healthcare utilization to understand the benefit of PRx within a healthcare system. Ultimately, if proven effective, the route of sustainability would be for federal policy to align and insurance coverage of PRx as medical interventions. A question remains, whether PRx interventions can be short-term interventions or whether they need to be sustained for a longer period of time to see long-term impacts. The goal may be to intervene during a window of opportunity, to expose, educate, and establish long-term healthy behaviors that can alter an individual’s trajectory of disease risk, putting them on a path of healthy lifestyle behaviors that will last a lifetime.

This study has some limitations. Eligible studies were limited to PRx interventions within a healthcare system, which excluded community and FNPs. While these are important programs, they were outside the scope of this review and have been described elsewhere.^18–20,77^ Search criteria were limited to the U.S. and excluded studies conducted before 2000. However, most studies were published within the last two years and have broad geographic range, making this scoping review timely and somewhat generalizable. Lastly, being a scoping review, the study was unable to measure aggregated outcomes and effect sizes across studies.

## Conclusion

The studies explored here show that PRx interventions may support household food security and improve nutritional knowledge and F/V intake in children and adult caregivers. While quantitative health metrics are currently lacking, longer-term evaluation of pediatric PRx outcomes will help identify the impact of these programs on health outcomes across the lifespan. Additional research in this field should continue to explore qualitative experiences while also incorporating more rigorous study designs, larger sample sizes, quantitative analyses of behavioral and health outcomes, cost-effectiveness assessments, and tracking of healthcare utilization. This work will require a multi-disciplinary approach that includes the family unit, community resources, the healthcare team, payers, and trained evaluators with standardized tools to offer the most useful information to promote the advancement and integration of PRx interventions.

## Data Availability

The datasets generated during and/or analyzed during the current study are available from the corresponding author on reasonable request.
